# Relationships of Biomass with Environmental Factors in the Grassland Area of Hulunbuir, China

**DOI:** 10.1371/journal.pone.0102344

**Published:** 2014-07-17

**Authors:** Miao Liu, Guohua Liu, Li Gong, Dongbo Wang, Jian Sun

**Affiliations:** 1 State Key Laboratory of Urban and Regional Ecology, Research Center for Eco-environmental Sciences, Chinese Academy of Sciences, Beijing, China; 2 University of Chinese Academy of Sciences, Beijing, China; 3 Key Laboratory of Ecosystem Network Observation and Modeling, Institute of Geographic Sciences and Natural Resources Research, Chinese Academy of Sciences, Beijing, China; 4 Monitoring Station for Eco-environment of Hulunbuir, Hulunbuir, China; Institute of Botany, Chinese Academy of Sciences, China

## Abstract

Many studies have focused on the relationship between vegetation biomass and environmental factors in grassland. However, several questions remain to be answered, especially with regards to the spatial pattern of vegetation biomass. Thus, the distributed mechanism will be explored in the present study. Here, plant biomass was measured at 23 sites along a transect survey during the peak growing season in 2006. The data were analyzed with a classification and regression tree (CART) model. The structural equation modeling (SEM) was conducted to explicitly evaluate the both direct and indirect effects of these critical environmental elements on vegetation biomass. The results demonstrated that mean annual temperature (MAT) affected aboveground biomass (AGB) scored at −0.811 (*P*<0.05). The direct effect of MAT on belowground biomass (BGB) was −0.490 (*P*<0.05). The results were determined by SEM. Our results indicate that AGB and BGB in semi-arid ecosystems is strongly affected by precipitation and temperature. Future work shall attempt to take into account the integrated effects of precipitation and temperature. Meanwhile, partitioning the influences of environmental variations and vegetation types are helpful in illuminating the internal mechanism of biomass distribution.

## Introduction

Vegetation biomass, which is usually illustrated by aboveground biomass (AGB), belowground biomass (BGB) and ratio of root to shoot (R/S), is regarded as an important parameter in indicating the carbon cycles of terrestrial ecosystems and in global climate models [1]–[3]. In particular, the majority of CO_2_ in the atmosphere was fixed by grassland ecosystems, which account for 1/4 of the land surface and 1/10 of global carbon storage [4], [5]. Changes in environmental conditions will produce rapid and profound influences on vegetation biomass [6], [7]. Furthermore, the spatial patterns of biomass shape the dynamics of the grassland’s carbon cycles [8]. Therefore, it is necessary to explore the mechanisms of how biomass distributed patterns respond to environmental variation and climate change [9]. Hulunbuir grassland, with one hundred thousand square kilometers of total grassland area, its large tracts of grassland make this area very suitable for research along the different ecological gradients.

The spatial pattern of vegetation biomass is severely shaped by environmental elements. There are many studies examining the relationship between vegetation biomass and environmental conditions. For instance, Peng et al. [10] found that net primary production responds non-linearly to increased vs. decreased rainfall in semi-arid grassland ecosystem, Inner Mongolia. Chang et al. [11] also documented that annual precipitation and air temperature are the key factors affecting the aboveground net primary productivity (ANPP) in temperate grasslands, and hold that ANPP increased with the increasing of precipitation, and declined with the air temperature. Fan et al. [12] reported that the proportion of belowground biomass increased as temperature decreased in Inner Mongolia, and was distributed more deeply in desert grassland owing to the aridity of the grassland types. Meanwhile, changes of AGB and BGB were examined in relation to gradients of temperature and precipitation. Total biomass was negatively correlation with temperature, but positively weak correlation with mean annual precipitation [13]. As a matter of fact, the complex change of biomass was caused by the meteorological factors and soil properties [14]. However, the studies regarding the magnitude of the concrete influence of the main environmental elements on vegetation biomass, especially along the gradients of precipitation and temperature across Hulunbuir grassland, remain few. The principal purposes of the present study were to explore the spatial distribution pattern of AGB, BGB and R/S along the gradients of precipitation and temperature using data investigated from 23 sites across Hulunbuir grassland; examine the relationships of AGB, BGB and R/S with the relevant environmental factors and to screen out the main factors using the classification and regression tree (CART) model; and to identify the concrete effect magnitude of the key elements on the AGB, BGB and R/S using Structural Equation Modeling (SEM). The final objective was to indicate the internal controlling factors of vegetation biomass along the gradients of precipitation and temperature.

## Materials and Methods

### Ethics Statement

For each site, no specific permits were required for the sample collected and the field studies did not involve endangered or protected species in Hulunbuir, Inner Mongolia.

### Study Area

The study area is situated in the western portion of Mt. Daxing’anling, Hulunbuir, Inner Mongolia, China (115°31′–126°04′E, 47°05′–53°20′N). The mean annual precipitation is 339 mm and the mean annual temperature is 2.2°C [15], [16]. The topographic features in the area are relatively changeless and the maximal difference in elevation is not more than 50 m. Chernozem and chestnut soil are the main soil types [17]. It is provided with broad precipitation and temperature gradients that are strongly related to the spatial patterns of grassland biomass in the Hulunbuir zone [18].

### Data Collection and Sample Analysis

**Figure 1:**
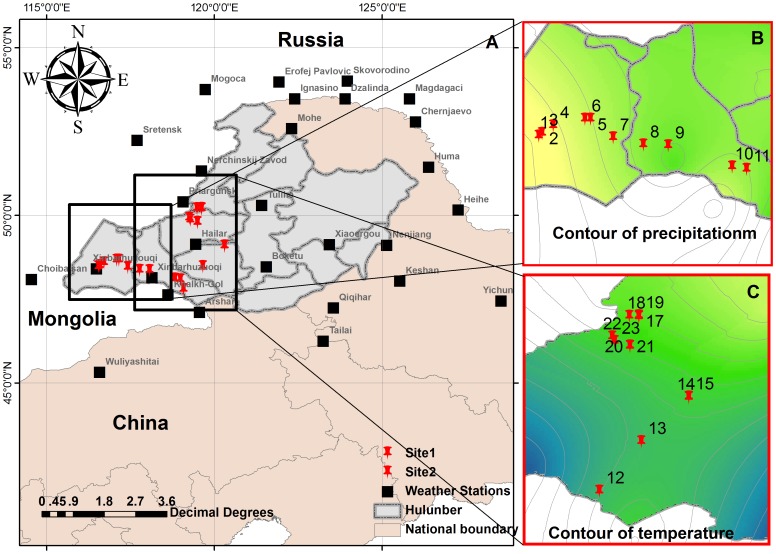
Spatial distribution of the sampling sites across the Hulunbuir grasslands, China. Samples (A) were collected along the gradients of precipitation (B) and temperature (C).

In 2006, 23 sites crossing from west to east and from south to north were surveyed in Hulunbuir grassland during the vegetation growth period (June, July and August). Sampling sites were established along the gradients of precipitation and temperature (Fig. 1). From each site, we harvested AGB and BGB in five plots (1 m×1 m) of similar topography and environmental conditions every 10 m intervals along a transect. AGB was determined by clipping the plants at ground level and oven-drying them at 65°C until they reached a constant weight. BGB was collected from soil depths of 0 cm to 30 cm, where most of the belowground biomass is located [19]. The root samples were obtained from the blocks using 5 cm diameter soil cores, soaked in water to remove the residual soil via a 0.5 mm sieve, and dried at 65°C to a constant weight. Meanwhile, soil samples were collected from five replicate soil profiles to determine soil properties at the 30 cm soil depth. After being air-dried and sieved (using 2 mm mesh), the soil samples were carefully handpicked to extract the surface organic materials and fine roots for soil chemical properties analysis. Each mixed soil sample was divided into two parts. One sub-sample was oven-dried at 105°C to a constant weight to measure gravimetric soil water content (SWC) and soil bulk density (BD). The remaining soil was ground in a ball mill for soil total organic carbon (TOC), total nitrogen (TN), total phosphorous (TP) and available phosphorous (AP) analysis. Soil properties were determined following all standard protocols [20]. Meteorological factors were obtained using spatially interpolated methods from the records of 28 weather stations (Fig. 1). Annual mean temperature (MAT)and annual mean precipitation (MAP) (2003–2006) were regarded as climate factors, and the data of longitude and latitude for each sampling site was determined using the Global Positioning System (GPS).

### Data Analysis

The entire document was analyzed according to the followings three steps. First of all, the data regarding MAP, MAT, TOC, TP, AP, TN, BD, SWC, AGB, BGB and R/S were handled through Spearman correlation analysis after normal distribution testing. Secondly, we used classification and regression tree (CART) [21] to screen out the crucial variables influencing vegetation biomass from all samples of environmental factors along gradients of precipitation and temperature according to the data collected across various studied sites. The CART method facilitates determination of the possible interactions and adjustments necessary for making decisions [22], as it can identify the critical variables that significantly influence the response variables. The specific operating process has been described in Sun’s report [8] and the R guideline book (R Development Core Team, 2011). Finally, structural equation modeling (SEM) [23] was conducted to explicitly evaluate both the direct and indirect effects of these critical environmental elements on vegetation biomass (AGB, BGB and R/S). SEM has been used in recent studies to exactly assess the causal relationships among multiple interacting variables [24]–[26]. In this study, statistical analysis and plotting were performed using the R software (version 2.15, R Development Core Team, 2011).

## Results

### Descriptive Statistic of AGB, BGB and R/S

**Figure 2:**
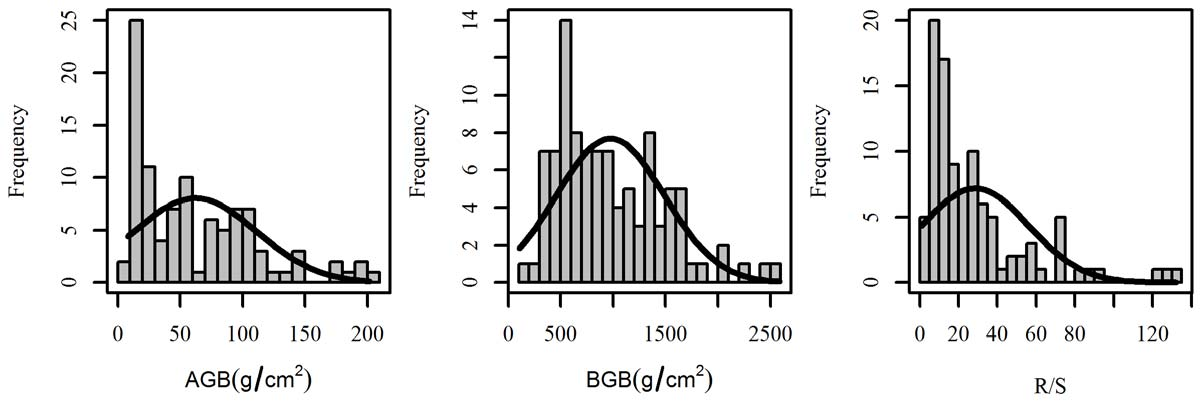
Frequency distribution curves of the AGB, BGB and R/S; the samples were collected across the Hulunbuir grasslands. All designations are the same as those in the footnotes below Table 1.

AGB, BGB and R/S all exhibited large variations along the sampled transect, ranging from 8.37 to 201.96 g m^–2^ for AGB, 105.99–2586.50 g m^–2^ for BGB and 1.01–131.98 for R/S (Fig. 2). The mean values were 62.01 g m^–2^, 974.21 g m^–2^ and 28.67 for AGB, BGB and R/S, respectively. The standard deviation values of AGB, BGB and R/S were 48.87, 513.72 and 27.46, in sequence.

### Correlationship of AGB, BGB and R/S with Environmental Factors

The Spearman correlation analysis method was adopted to analyze the correlation of AGB, BGB and R/S with environmental factors (Table 1). The results demonstrated that AGB was positively related to MAP, TOC and TN across the sampling sites, with correlation coefficients of 0.562, 0.613 and 0.511, respectively. By contrast, AGB was negatively related to MAT, AP and BD, with correlation coefficients of 0.661, 0.579 and 0.650, respectively, while its relation with SWC and TP was non-significant. BGB was positively related to the MAP, TOC and TN at the 0.05 level, with correlation coefficients of 0.406, 0.434 and 0.359, respectively. The relationships of BGB with SWC, TP, AP and BD were non-significant. Meanwhile, R/S was positively related to AP and BD, with correlation coefficients of 0.446 and 0.557, while it had negative correlations to MAP, TOC and TN, with correlation coefficients of −0.328, 0.368 and 0.312 (*P*<0.05). The relationships of R/S with SWC and AP were non-significant.

**Table 1:** Coefficient of correlation between biome and environmental factors (Spearman).

	MAP	MAT	TOC	TP	AP	TN	BD	SWC	AGB	BGB	R/S
MAP	1										
MAT	−0.923**	1									
TOC	0.610**	−.682**	1								
TP	−0.010	−.053	0.473**	1							
AP	−0.618**	.619**	−0.259**	0.331**	1						
TN	0.537**	−.600**	0.915**	0.553**	−0.185	1					
BD	−0.555**	.638**	−0.763**	−0.417**	0.437**	−0.783**	1				
SWC	−0.034	.147	0.621**	0.396**	0.165	0.620**	−0.312	1			
AGB	0.562**	−.661**	0.613**	−0.076	−0.579**	0.511**	−0.650**	0.167	1		
BGB	0.406**	−.429**	0.434**	0.139	−0.179	0.359**	−0.272	−0.097	0.255*	1	
R/S	−0.328**	.421**	−0.368**	0.138	0.446**	−0.312**	0.557**	−0.139	−0.826**	0.306**	1

Note: **Correlation is significant at the 0.01 level.*Correlation is significant at the 0.05 level (2-tailed). MAP (Mean annual precipitation), MAT (Mean annual temperature), TOC (Total organic carbon ), TP (Total phosphorous), AP (Available phosphorous ), TN (Total nitrogen ), BD (Soil bulk density), SWC (Soil water content), AGB (Aboveground biomass), BGB (Belowground biomass ) and R/S (the ratio of root and shoot). The sample size is 100.

### Identification of Critical Factors by the CART Model

**Figure 3:**
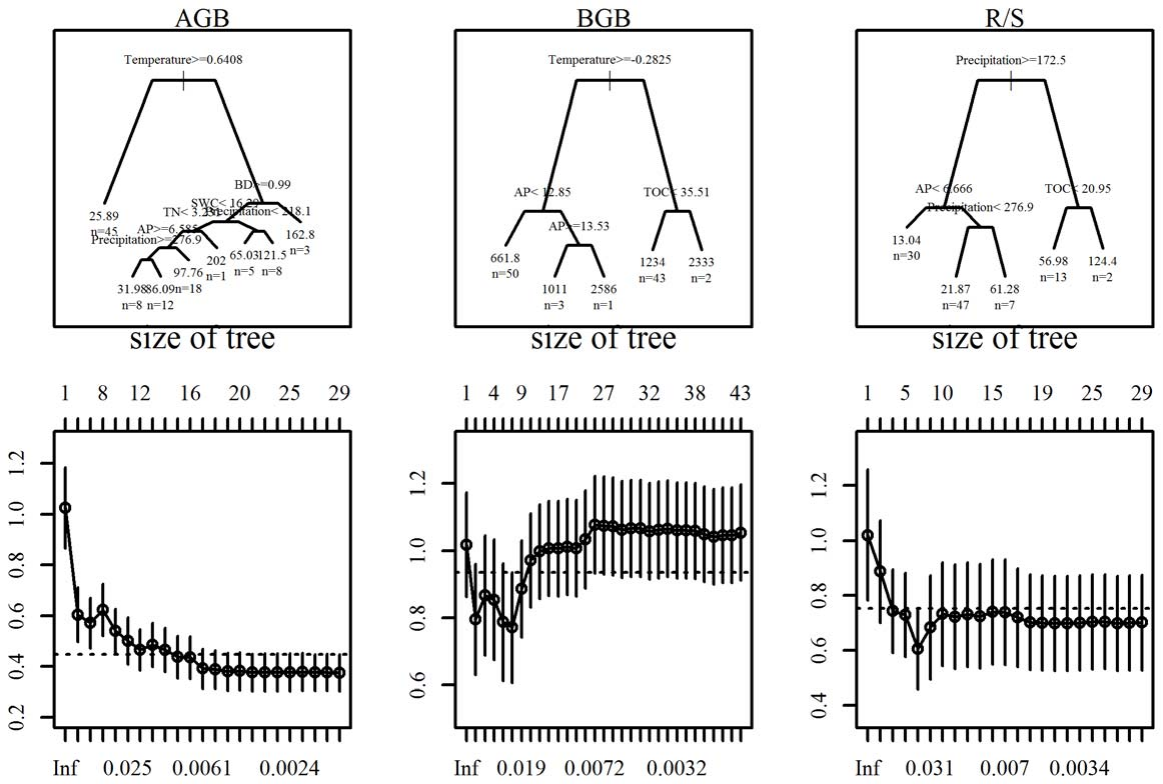
CART analyses of the relationships between biome and environmental factors along the gradients of precipitation and temperature in the Hulunbuir grasslands. The key environmental factors were screened in panels A (AGB), B (BGB) and C (R/S). Branches are labeled with criteria used to segregate data. Values in terminal nodes represent mean vegetation biomass of sites grouped within the cluster. n = number of plots in the category. The below corresponding panels were structural complexity (cp value) of trees. All designations are the same as those in the footnotes below Table 1.

The impacts of all kinds of environmental factors on vegetation biomass (AGB, BGB and R/S) were observed using the CART model. The optional tree was developed. As shown in Fig. 3A, the analysis indicated that MAP, MAT, AP, TN, SWC and BD are most closely associated with large-scale variations in AGB. For the second tree (Fig. 3B), three critical environmental factors containing MAT, AP and TOC were obtained, having a significant influence on BGB. For the third tree (Fig. 3C), R/S was influenced mainly by MAP, TOC and AP.

### Structural Equation Modeling to Explain the Effects of Environmental Variables on AGB, BGB and R/S

The critical variables (MAP, MAT, AP, TN, SWC and BD) were inter-correlated, and these apparent relationships combined both direct and indirect correlations (Fig. 3). Thus, we further used SEM to explicitly evaluate the causal relationships among these interacting variables.

**Figure 4:**
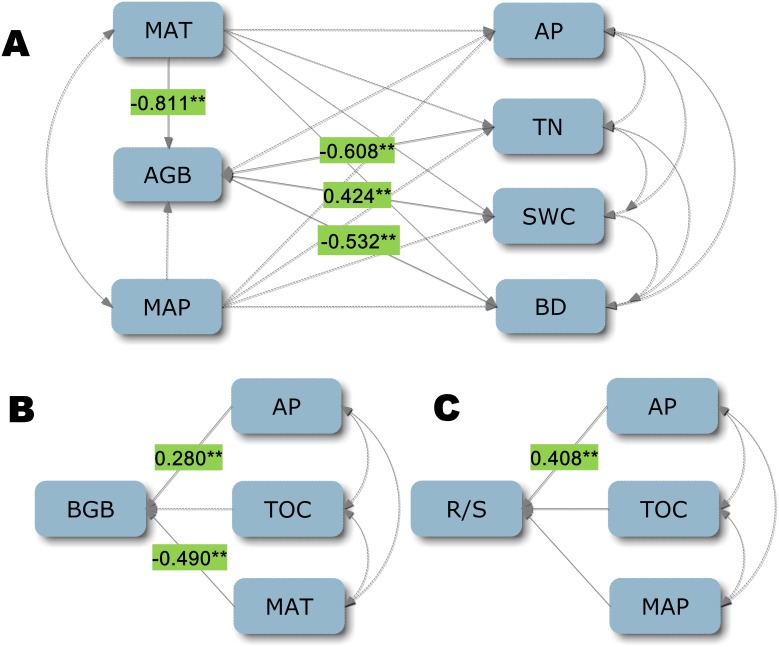
The establishment of a structural equation model for A) aboveground biomass (AGB), B) belowground biomass (BGB) and C) root to shoot ratio (R/S). Each line represents a direct linear causal relationship. The arcs show the correlation between two variables. Values on lines are path coefficients. The asterisks are significant at *P* = 0.05 level. The coefficients that are not statistically significant are shown by dashed arrows. All values are standardized. All designations are the same as those in the footnotes below Table 1.

From the SEM (Fig. 4), it is evident that, MAT, TN, SWC and BD affected AGB (direct effect), scored at −0.811 (*P*<0.05), −0.608, 0.424 and 0.532, respectively (Table 2, Fig. 4A). The direct effects of AP and MAT on BGB were 0.280 and −0.490 (*P*<0.05), respectively (Table 2, Fig. 4B). AP had significantly positive direct effects on R/S at 0.408 (Table 2, Fig. 4C). The rank of total effects on AGB, in decreasing order, was: TN, MAT, SWC, BD and MAP. MAT had the strongest negative effects on both AGB and BGB among all predictors (Table 2, Figs. 4A, B), whereas no significant effect of MAP on AGB was found. AGB and BGB responded similarly to MAT. The standardized total effect of MAT on AGB was −0.381, consisting of direct effects (path coefficient = −0.811, *P*<0.05) and indirect effects through soil variables (path coefficient = 0.43). Among soil variables, TN and BD had significant negative effects on AGB, with path coefficients of −0.608 and −0.532 (Table 2, Fig. 4A), whereas SWC had a positive effect on AGB (path coefficient = 0.424). The total effect of AP on R/S was 0.389, consisting of direct effects (path coefficient = 0.408, *P*<0.05) and indirect effects through soil variables (path coefficient = −0.019). However, neither the direct nor indirect effects of MAP on R/S were significant (Table 2, Fig. 4C).

**Table 2:** Standardized direct effect, indirect effect and total effect of critical environmental factors on aboveground biomass (AGB), belowground biomass (BGB) and root to shoot ratio (R/S).

Endogenous variable	Exogenous variable	Direct effect	Indirect effect	Total effect
AGB	MAP	−0.047	−0.028	−0.075
	MAT	−0.811**	0.430	−0.381
	AP	0.097	−0.277	−0.180
	TN	−0.608**	0.156	−0.452
	SWC	0.424**	−0.111	0.323
	BD	−0.532**	0.261	−0.271
BGB	MAT	−0.490**	0.056	−0.434
	AP	0.280**	0.01	0.291
	TOC	0.062	0.052−	0.114
R/S	MAP	−0.049	0.183	0.134
	AP	0.408**	−0.019	0.389
	TOC	−0.142	0.055	−0.087

Note: The “**” represented the significant. All designations are the same as those in the footnotes below Table 1.

## Discussion

### Characteristics of Vegetation Biomass across all Samples in the Temperate Grassland

The values of vegetation biomass exhibited large variations along the sampled transect. The mean value of AGB in the present study was found to be lower than that in the temperate grasslands of Inner Mongolia (135.3 g m^–2^) [27]. By contrast, BGB and R/S in the temperate grassland of Hulunbuir was found to be higher than it is in the temperate grasslands of Inner Mongolia (775.2 and 6.3 g m^–2^). Meanwhile, BGB was far larger than AGB across Hulunbuir grassland, which was approximately 15 times greater than AGB. This result indicated that vegetation biomass in the temperate grassland of Hulunbuir was present to a greater degree below ground than above. Similar results were detailed in Ma’s report [27], which described how BGB was approximately 6 times greater than AGB in the temperate grasslands of Inner Mongolia. The average value of R/S in Hulunbuir moderate grassland is far larger than that observed in global temperate grasslands (4.2) [1]. The higher R/S in Hulunbuir grasslands might be associated with the comparatively slow depletion of carbohydrates in roots, resulting from low respiration rates in the extremely cold winters there, and might also be due to slower root turnover in the colder environment relative to the mean level of the global temperate grasslands [28]. In addition, R/S values found in the present study ranging from 1.01 to 131.98 were consistent with results reported by Yang et al. [13], who performed a field investigation from 2001 to 2004, discovering that R/S values have the potential to vary greatly as a result of climate change and anthropogenic activities [29]–[31]. For example, long term grazing in grasslands may result in the reduction of AGB and ultimately higher R/S.

### Effect of critical factor on Spatial Pattern of AGB, BGB and R/S

Moreover, based on the Spearman correlation analysis between biomass and environmental factors, we found that most environmental factors showed a significant correlation at 0.05 levels (Table 1). This may be a result of the degree of multicollinearity with respect to the effect of environmental factors on vegetation biomass, consistent with Sun’s report [8].

We used the CART model and SEM [23] as new approaches to conduct variable selection and quantify its effect so as to screen out the critical factors associated with AGB, BGB and R/S and then to identify direct and indirect factors while determining the extent to which these factors may constrain vegetation biomass. To our knowledge, the efficiency of these approaches has not been evaluated empirically in vegetation biomass research simultaneously. Traditionally, stepwise selection and linear regression are used to identify and rank the limiting factors in grassland biomass studies. However, when performing stepwise selection, closely covariate parameters cannot be selected simultaneously in the final model, because the explanatory power does not increase when a closely related variable is included [32]. Field studies examining ecosystem responses to climatic and other environmental changes typically use naturally occurring climatic gradients. Nevertheless, Burke et al. [33] raised the issue that there are inherent problems with utilizing simple statistical relationships of spatial variability as foundations for understanding ecosystem function, because complex covariance along the gradient occurs across large spatial scales, leading to the problem that actual and apparent controlling factors may be confounded. SEM, however, is an appropriate option [34]. The quantitative procedure in the current study demonstrated that the strongest direct factors influencing AGB at the regional scale were MAT, TN, SWC and BD. While the largest total element impacting R/S was AP, AP and MAT both had major direct influences on BGB. This holistic approach is appropriate in across-site comparisons of ecosystem structure and function. Additionally, we discovered that longitude and latitude, which indicated the gradient of precipitation and temperature accordingly, were almost the main determinants on biome with a comparatively larger magnitude of total effect than that of all other relevant environmental factors. Therefore, it is necessary to explore the mechanism of biomass distribution along the gradient of precipitation and temperature.

Precipitation and temperature are considered to be the limiting factors for the growth and distribution of vegetation over the long term [35]. Previous studies precipitation has large impacts on grassland ecosystems biomass in Inner Mongolia [11], [27] However, in the present study, AGB and BGB showed a decreasing trend with increasing temperature (Fig. 4A, B), while R/S demonstrated a reverse change (Table 1). The spatial pattern of vegetation biomass in Inner Mongolia in this dry area is associated with temperatures [13]. Under drought conditions high temperatures may further restrain plant photosynthesis. This was shown by Xu and Zhou [36], who reported that high temperatures resulted in the reduction of photosynthetic rate and biomass of *L. chinensis* steppe. Furthermore, higher temperatures will result in increased evaporation, intensifying drought and reducing biomass [11].

In our case, when MAP is retained in the model, MAT will not be selected. Therefore, our results showed temperature to be a significant influence on AGB and BGB, while precipitation was not shown in Fig. 4. As a matter of fact, precipitation also had the strongest positive effect on AGB and BGB (Table 1). This phenomenon indicates that there is an integrated effect between temperature and precipitation on plant growth [37]. Our findings reinforce the idea that precipitation is major limiting factor that control the functions of ecosystems in terrestrial biomes, particularly in arid and semi-arid ecosystems [38]–[41]. In addition, we found that TOC and TN are also positively related to precipitation [42] and negatively related to temperature (Table 1). Kirschbaum [43] holds that warming will have the effect of reducing soil organic carbon by stimulating decomposition rates more than NPP. On the other hand, some scholars hold the opposite view [44], suggesting that adequate precipitation and temperatures that are not too high can be used to create good soil nutrient conditions for plant growth [33]. Relevant to the above statement, the spatial pattern of vegetation biomass was influenced by soil nutrient level (Fig. 4B, C), and soil nutrient levels were affected by the conditions of water and heat. Soil nutrient levels can be improved by more litter being returned to soil resulting from more plant biomass with the addition of well water and ideal heat conditions.

Soil nitrogen has been found to be a limiting factor for AGB and BGB in most terrestrial ecosystems [37], [45]. Nevertheless, others have reported limited or no influence of soil nitrogen on ecosystem production [46], [47]. In the present study, we found that there was negative relationship of AGB with total nitrogen content. Thus, we concluded that the different responses of plants to nitrogen content are a result of different nitrogen deficiency levels of the local systems [37]. Soil phosphorous, generally regarded as the most critical factor for plant growth [48], the biomass was related with available phosphorous (Table 1, Figure 4). Meanwhile, the previous studies suggested that soil water content play an important role on biomass allocation [49], and the soil physical structure (e.g soil bulk density) might be more important for determining BGB than other factors [50].

## Conclusion

Our results indicate that precipitation and temperature strongly affect aboveground and belowground biomass in semi-arid ecosystems. Future work shall attempt to take into account the integrated effects of precipitation and temperature, which could possibly explain the spatial variance of AGB, BGB and R/S better than MAP or MAT alone. Meanwhile, partitioning the influences of environmental variations and vegetation types is helpful in illuminating the internal mechanism of biomass distribution along the gradient of precipitation and temperature.
